# Travel-related MERS-CoV cases: an assessment of exposures and risk factors in a group of Dutch travellers returning from the Kingdom of Saudi Arabia, May 2014

**DOI:** 10.1186/1742-7622-11-16

**Published:** 2014-10-17

**Authors:** Ewout B Fanoy, Marianne AB van der Sande, Marleen Kraaij-Dirkzwager, Kees Dirksen, Marcel Jonges, Wim van der Hoek, Marion PG Koopmans, Douwe van der Werf, Gerard Sonder, Charlie van der Weijden, Jet van der Heuvel, Luc Gelinck, Jolande W Bouwhuis, Arianne B van Gageldonk-Lafeber

**Affiliations:** 1 National Institute for Public Health and the Environment, Centre for Infectious Disease Control, Bilthoven, The Netherlands; 2 European Programme for Intervention Epidemiology Training (EPIET), European Centre for Disease Prevention and Control, (ECDC), Stockholm, Sweden; 3 Public Health Service region of Utrecht, Zeist, The Netherlands; 4 Julius Center, University Medical Center Utrecht, Utrecht, The Netherlands; 5 Public Health Service Haaglanden, Den Haag, The Netherlands; 6 Erasmus Medical Center, Rotterdam, The Netherlands; 7 Public Health Service IJsselland, Zwolle, The Netherlands; 8 Public Health Service Amsterdam, Amsterdam, The Netherlands; 9 Public Health Service Flevoland, Lelystad, The Netherlands; 10 Public Health Service Hart voor Brabant’s, Hertogenbosch, The Netherlands; 11 Medical Centre Haaglanden, The Hague, The Netherlands; 12 Isalaklinieken, Zwolle, The Netherlands; 13 National Institute for Public Health and the Environment, Epidemiology and Surveillance Unit, Antonie van Leeuwenhoeklaan 9, Bilthoven, MA 3721, The Netherlands

**Keywords:** Middle East respiratory syndrome coronavirus, MERS, MERS-CoV, Exposure, Risk factors, Epidemiology

## Abstract

**Background:**

In May 2014, Middle East respiratory syndrome coronavirus (MERS-CoV) infection, with closely related viral genomes, was diagnosed in two Dutch residents, returning from a pilgrimage to Medina and Mecca, Kingdom of Saudi Arabia (KSA). These patients travelled with a group of 29 other Dutch travellers. We conducted an epidemiological assessment of the travel group to identify likely source(s) of infection and presence of potential risk factors.

**Methods:**

All travellers, including the two cases, completed a questionnaire focussing on potential human, animal and food exposures to MERS-CoV. The questionnaire was modified from the WHO MERS-CoV questionnaire, taking into account the specific route and activities of the travel group.

**Results:**

Twelve non-cases drank unpasteurized camel milk and had contact with camels. Most travellers, including one of the two patients (Case 1), visited local markets, where six of them consumed fruits. Two travellers, including Case 1, were exposed to coughing patients when visiting a hospital in Medina. Four travellers, including Case 1, visited two hospitals in Mecca. All travellers had been in contact with Case 1 while he was sick, with initially non-respiratory complaints. The cases were found to be older than the other travellers and both had co-morbidities.

**Conclusions:**

This epidemiological study revealed the complexity of MERS-CoV outbreak investigations with multiple potential exposures to MERS-CoV reported such as healthcare visits, camel exposure, and exposure to untreated food products. Exposure to MERS-CoV during a hospital visit is considered a likely source of infection for Case 1 but not for Case 2. For Case 2, the most likely source could not be determined. Exposure to MERS-CoV via direct contact with animals or dairy products seems unlikely for the two Dutch cases. Furthermore, exposure to a common but still unidentified source cannot be ruled out. More comprehensive research into sources of infection in the Arabian Peninsula is needed to strengthen and specify the prevention of MERS-CoV infections.

## Background

In 2012, the Middle East respiratory syndrome coronavirus (MERS-CoV) was isolated for the first time from the sputum of a 60-year-old man who presented with an acute pneumonia in Kingdom of Saudi Arabia (KSA) [1]. As of June 24 2014, 707 laboratory-confirmed cases of MERS-CoV infection have been reported to the WHO including 252 (36%) fatal cases [2]. Following the first upsurge of cases in spring 2013, a second wave occurred in 2014, mainly in KSA [3]. Most cases so far either resided in or travelled to the Arabian Peninsula and neighbouring countries or were close contacts of these cases. Furthermore, travel related cases have been reported from the United States of America (USA), the United Kingdom (UK), Germany, France, Tunisia, Italy, Greece, Algeria, Egypt, the Philippines and Malaysia [3-7].

So far, mainly sporadic cases are reported as well as a few hospital outbreaks, which is in line with a low reproduction rate (R0) of MERS-CoV, estimated to range from 0.4 to 1.5 [8-10]. The median incubation period is estimated to be 5.2 days (95% CI 1.9-14.7 days) [8]. For most non health-care associated cases no clear source of infection could be identified, although camels are considered a reservoir as the virus and antibodies against MERS-CoV have been identified in camels (*Camelus dromedarius*) and in their milk [9,10]. The exact modes of transmission of the virus to humans remain unclear [11-14], leaving the possibility for other yet unidentified sources of infection. To stop or reduce transmission of MERS-CoV and prevent new human infections, it is important to identify all potential sources of infections as well as risk factors and route(s) of transmission.

In May 2014, MERS-CoV infection was diagnosed in two Dutch residents. These cases travelled with a group of 29 other people, returning to the Netherlands from pilgrimage to Medina and Mecca, KSA [15]. We conducted a comprehensive epidemiological assessment of the travel group aiming to identify the likely source(s) of infection and presence of potential risk factors for these two cases.

## Methods

We compiled a questionnaire based on sample questionnaires originating from two WHO-protocols aimed to asses risk factors and investigate contacts of patients with MERS‐CoV infection [16,17]. We adapted the questionnaire to the specific route and activities of the Dutch travellers. Our questionnaire covered human, animal and food exposures to MERS-CoV during the whole trip to KSA. The questionnaires consisted of 28 open and closed questions, and it took approximately 20 minutes to complete the face-to-face interview with the travellers (Additional file 1). Nurses of the public health services administered the questionnaires approximately 3 weeks after return to the Netherlands. For the two cases the questionnaire was completed with data on the travel route and stay in KSA collected by the National Coordination Centre for Communicable Disease Control.

## Results

The patients were a 70-year-old man (Case 1) and his 73-year-old sister (Case 2), both having underlying cardiovascular co-morbidities and diabetes mellitus. The remaining travel group included 29 persons (41% male) aged between 10 and 70 years (median age: 55 years). Seventeen (59%) of the non-cases did not report any co-morbidities (Table 1). Upon identification of Case 1, throat swabs of all contacts were tested by PCR to assess other MERS-CoV cases. Apart from Case 2, who had reported mild respiratory complaints starting on the 5^th^ of May (t = day 9), all other 29 travellers tested negative and did not develop symptoms.

**Table 1:** Self-reported co-morbidity for the 31 persons who made a pilgrimage to Medina and Mecca, Kingdom of Saudi Arabia, April-May 2014

**Co-morbidity**	**MERS-CoV patients N = 2 n (%)**	**Other travellers N = 29 n (%)**
Cardiovascular diseases	2 (100)	4 (14)
Diabetes mellitus	2(100)	6 (21)
Other co-morbidity*	0 -	9 (31)
No com-morbidities	0 -	12 (41)

*Mainly allergy reported.

**Table 2:** **Overview of potential MERS-CoV exposures of cases and asymptomatic travellers during a visit to the Kingdom of Saudi Arabia, April 26**^**th**^**- May 10**^**th**^**2014**

	**Case 1**	**Case 2**	**Asymptomatic travellers (n = 29)**
**Animal exposure**			
Visit to camel farm	No	No	14 (48%)
Direct contact with camels	No	No	4 (14%)
Indirect contact with camels	No	No	8 (28%)
**Food exposures**			
Consumption of unpasteurized milk	No	No	11 (38%)
Visit to local market in Medina	Yes	No	21 (72%)
Consumption of fruits on market	No	No	7 (24%)
Buying souvenirs on market	No	No	14 (28%)
**Human exposures**			
Contact with coughing patients (excluding case 1 and 2)	Yes, in waiting room of a hospital	No	2 (7%)
Hospital visit (due to other non-MERS related illnesses)	Yes	No	1 (3%)
Minimal social contact with case 1 and/or 2	Case 1 and 2 had daily contact with each other		17 (59%)
Daily contact with case 1 and/or 2	Case 1 and 2 had daily contact with each other and shared hotel rooms		12 (41%)

MERS-CoV: Middle East Respiratory Syndrome coronavirus.

### Travel route exposures

**Figure 1:**
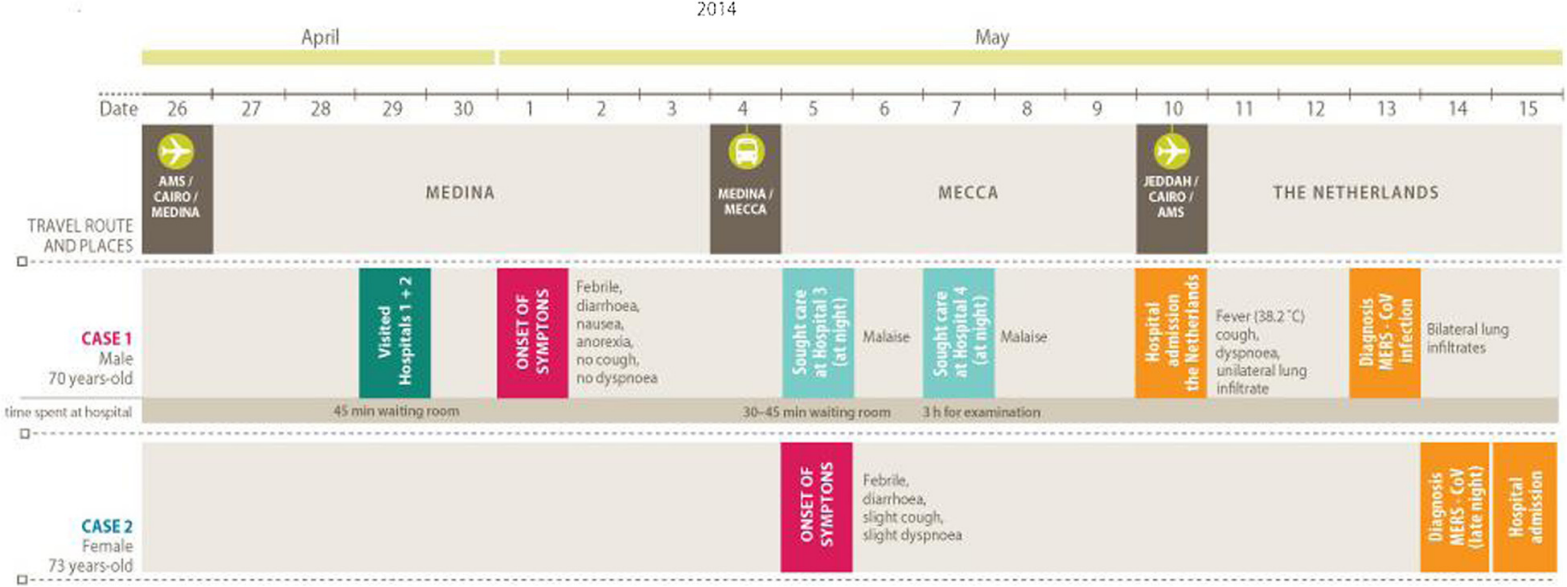
**Timeline of events for two MERS-CoV patients returning to the Netherlands from Saudi Arabia, May 2014.** This is a figure created by Rita de Sousa and published in the following article in *Eurosurveillance*: Kraaij – Dirkzwager M, et al. [15].

The group made a pilgrimage to Medina and Mecca, KSA (Figure 1). They arrived on the 26th of April 2014 (day 0) in Medina, left by private bus to Mecca on the 4^th^ of May (day 8) and returned to Amsterdam on the 10^th^ of May (day 14). Case 1 started to feel feverish and had diarrhoea on the 1^st^ of May (day 5). Respiratory complaints started on the 10^th^ of May (day 14). MERS-CoV was identified in Case 1 on the 13^th^ of May (day 17) and in Case 2 on the 14^th^ of May (day 18). Detailed case reports of the two patients are described by Kraaij et al. [15]. During their pilgrimage, the whole group stayed in the same hotels and had breakfasts together. There was no fixed collective travel programme, but they had some joint visits to several mosques. The travellers also spent time alone or in smaller subgroups, visiting different mosques, markets and restaurants.

The exposures for both the cases and 29 asymptomatic travellers during their visit to Saudi Arabia are summarized in Table 2.

### Animal exposure

On the 3^rd^ of May (day 7), 12 travellers (excluding the two cases) made a trip to the touristic valley Wadi-e-Jinn, and stopped when they came across a herd of camels laying behind an improvised razor fence. Four travellers reported direct contact with the camels. Eight travellers mentioned indirect contact, for example via the fences surrounding the animals. Eleven travellers consumed unpasteurized camel milk, offered to them by the caretakers of the camels. Besides contact with camels, two travellers reported that they spotted many pigeons in the cities and fed them. No other direct contact with animals was mentioned.

### Food exposure

As mentioned above, eleven travellers, excluding the two cases, consumed unpasteurized camel milk. Furthermore, 22 travellers, including Case 1, visited one or more local markets in Medina. Seven of them, excluding Case 1, bought and consumed a range of fruits such as prunes and dates. Furthermore, 14 travellers bought souvenirs, such as dates, perfume and clothing on these markets.

### Human exposures

#### *Hospital exposures*

Case 1 and his son visited two hospitals in Medina on the 29^th^ of April (day 3) because of eye complaints of the son. As mentioned before, they spent 45 minutes in a crowded waiting room of a general hospital where they were exposed to coughing patients. Subsequently, the son was referred to a specialized eye clinic where they spent no time in a waiting room and did not notice any coughing patients.

On the 5^th^ of May (day 9), Case 1, accompanied by his son, visited an emergency department of a general hospital in Mecca because of acute malaise, weakness, nausea and diarrhoea (without respiratory complaints). These symptoms started on May 1 (day 5). Case 1 and his son spend 30-45 minutes in the waiting room, though they had no specific exposure to coughing people. On the 7^th^ of May (day 11) Case 1 returned to another hospital in Mecca, accompanied by his son, where he only briefly stayed in the waiting room, received antibiotic treatment and was observed for approximately three hours. Four other travellers, including his son, accompanied him. Two of them did not notice any coughing persons, while the other two did not answer this question. Case 2 did not visit a hospital during the pilgrimage or prior to her diagnosis in the Netherlands.

#### *Contact with coughing persons*

Two travellers (Case 1 and his son) waited 45 minutes in a crowded waiting room of a general hospital in Medina (29th of April, day 3) where they were exposed to coughing patients. One other traveller, who did not enter a hospital, reported coughing persons outdoors. The duration and intensity of this contact was not clarified in more detail. Case 1 and 2 had been in close contact with each other during the entire trip, as they shared hotel rooms.

All 29 travellers had been in contact with both cases during the trip. Seventeen travellers had minimal social contact with the two cases, for example only brief eye contact in the hotel lobby or in the bus. Twelve travellers reported daily contact, such as visits to the hotel room of Case 1 while he was sick, or accompanying him while shopping. The son of Case 1 had the most intensive contact sharing a hotel room with both cases. Nevertheless, his throat swabs tested negative to MERS-CoV by PCR during the follow-up after the identification of Case 1.

## Discussion

We found no indications that the two cases were infected via contact with specific animals or dairy products. Infection during a hospital visit could be a source for Case 1. Sequence analysis of parts of the viral genome collected from both patients had shown that the strains were nearly identical, suggesting that the cases have infected each other, or that they were exposed to a common source. In the latter case, exposure during a hospital visit is unlikely because Case 2 did not visit a hospital before onset of disease. The possibility of asymptomatic infections and transmission among the accompanying travellers cannot be ruled out, but seems unlikely in view of the negative PCR results. Subsequently, although considered unlikely, we cannot exclude the possibility of asymptomatic travellers being a source of infection for Case 1 and/or 2. No other common sources were identified although we cannot exclude the possibility of lack of accurate recall or indirect exposure to animal excreta. It might be possible that one or both cases were infected during non-specific contacts with the local population or the environment. If initial zoonotic introductions of MERS-CoV from camels have been followed by low level presence among the (asymptomatic) population or in the environment at large, this might result in an ongoing community-based outbreak, which, unlike SARS, cannot be controlled by rigorous hospital hygiene alone.

Both patients were of older age and had comorbidities. In general, older age and comorbidity are considered risk factors for symptomatic MERS-CoV infections, although this was not the case in all outbreaks [18,19]. In this study, the distribution of symptoms towards higher age and comorbidity fits this assumption. Ongoing serological analysis upon travellers and people exposed to the case in the Netherlands may reveal to what extent asymptomatic infection has occurred. The remainder of the travellers did not develop symptomatic MERS-CoV and did not have positive PCR-test results despite exposure to camels, dairy products and the two cases. This could point to a low transmissibility of the virus, which is also illustrated by the limited number of secondary infections among contacts of import cases described elsewhere, with the reservation that asymptomatic infections among the remaining travellers cannot be excluded fully as final serologic results are not available [5,6]. Currently, there is only limited epidemiological information published considering potential sources and transmission dynamics of MERS-CoV infection within the Arabian Peninsula. Descriptive studies among a well-defined group of travellers can therefore provide relevant epidemiologic information, even when the number of travellers is too small to draw definite conclusions. More in depth epidemiological investigation of comparable travel groups will add to the body of evidence, although the diversity of activities during a relatively short trip makes accurate recall of potential exposure challenging. Alternatively, prospective cohort studies among travellers to affected areas, whereby travellers are asked to keep a daily diary, are likely to be more informative and complete. Due to the uncertainty about the source, the detailed questions of the WHO-questionnaire were a very useful guide to decide on the most relevant exposures to be explored, adapted to the specific route of travel.

## Conclusions

The exact source of infection remains difficult to identify. Case 1 could have been infected during his visit to a hospital and subsequently have infected Case 2. However, exposure to a common source for both Case 1 and 2 cannot be ruled out. Besides, Case 1 may have been exposed to a yet unidentified source and subsequently have infected Case 2, or vice versa. More research to relevant sources in the Arabian Peninsula is needed. The suggested role of underlying disease to develop MERS-CoV infection is in line with the age and comorbidity of the two Dutch cases and the absence of symptomatic MERS-CoV infections among the younger and healthier travellers.

## Competing interests

The authors declare that they have no competing interests. All readers have read and approved the manuscript.

## Authors’ contributions

EF compiled the questionnaires, analysed the data and drafted the manuscript. MS is in charge of the epidemiology department; she designed and coordinated the study and reviewed the manuscript. MK assessed travel data and reviewed the manuscript. KD coordinated interviews with travellers and reviewed the manuscript. MJ was responsible for laboratory testing and reviewed the manuscript. WH is in charge of respiratory epidemiology department and reviewed the manuscript. MPGK is involved in laboratory testing and reviewed the manuscript. DW coordinated interviews with travellers and reviewed the manuscript. GS coordinated interviews with travellers and reviewed the manuscript. CW coordinated interviews with travellers and reviewed the manuscript. JH coordinated interviews with travellers and reviewed the manuscript. LG was involved in clinical care and interviewed a case. JB was involved in clinical care and interviewed a case. AG was leading the study, including the study design and analysis and she reviewed the manuscript. All authors read and approved the final manuscript.

## Authors’ informations

**Members of the MERS-CoV outbreak investigation team of The Netherlands** (in alphabetical order): Christel Bank (Medical Centre Haaglanden); Kees Dirksen (Public Health Service The Hague); Willem Geerlings (Medical Centre Haaglanden); Luc Gelinck (Medical Centre Haaglanden); Paul Groeneveld (Isala Klinieken); Bart Haagmans (Erasmus MC); Casper Jansen (Medical Centre Haaglanden); Marcel Jonges (RIVM Centre for Infectious Disease Research, Diagnostics and Screening); Michiel Knaven (Medical Centre Haaglanden); Marion Koopmans (Erasmus MC and RIVM Centre for Infectious Disease Research, Diagnostics and Screening); Marleen Kraaij – Dirkzwager (RIVM National Coordination Centre for Communicable Disease Control); Eliane Leyten (Medical Centre Haaglanden); Johan Mutsaers (Medical Centre Haaglanden); Suzan Pas (Erasmus MC); Stalin Raj (Erasmus MC); Chantal Reusken (Erasmus MC); Hella Smit (RIVM Communication); Rita de Sousa (RIVM Centre for Infectious Disease Research, Diagnostics and Screening and The European Programme for Public Health Microbiology Training (EUPHEM), European Centre for Disease Prevention and Control (ECDC)); Corien Swaan (RIVM National Coordination Centre for Communicable Disease Control); Ingeborg Thurkow (Public Health Service Ijsselland); Aura Timen (Chair, RIVM National Coordination Centre for Communicable Disease Control); Anouk Urbanus (RIVM National Coordination Centre for Communicable Disease Control); Paul van Beek (RIVM National Coordination Centre for Communicable Disease Control); Douwe van der Werf (Public Health Service IJsselland); Annemiek van der Eijk (Erasmus MC); Rianne van Gageldonk-Lafeber (RIVM Centre for Infectious Diseases, Epidemiology and Surveillance); Erik Verschuren (RIVM National Coordination Centre for Communicable Disease Control); Johan Versteegen (Public Health Service The Hague); Suzanne M. Witteveen-Pronk (Public Health Service IJsselland); Caroline Wortman (Medical Centre Haaglanden); Harald Wychgel (RIVM communication).

## Supplementary Material

Additional file 1 The questionnaire. Click here for file
