# Supplementary material for: Travel-related MERS-CoV cases: an assessment of exposures and risk factors in a group of Dutch travellers returning from the Kingdom of Saudi Arabia, May 2014
Source: Emerg Themes Epidemiol. 2014 Oct 17;11:16. doi: 10.1186/1742-7622-11-16 (PMC4200475; doi:10.1186/1742-7622-11-16)
Supplement: Additional file 1 — The questionnaire. [file 1742-7622-11-16-S1.docx]

Geachte heer, mevrouw,

Recent heeft u een reis gemaakt naar Saoedi Arabië. Binnen uw reisgezelschap zijn twee personen ziek geworden na besmetting met het MERS-coronavirus (MERS-CoV). MERS-CoV kan zeer ernstige luchtwegklachten veroorzaken. Inmiddels zijn er wereldwijd meer dan 570 patiënten met een bewezen MERS-infectie gemeld. De bron van infectie is nog niet duidelijk. Op verzoek van de Wereldgezondheidsorganisatie (WHO) is het Rijksinstituut voor Volksgezondheid en Milieu (RIVM) met de GGD een onderzoek gestart. De resultaten van dit onderzoek kunnen bijdragen aan de kennis over hoe mensen geïnfecteerd worden met MERS-CoV. Daarom is bij u al een keelwat en bloed afgenomen voor onderzoek. Uw antwoorden op de vragenlijst helpen bovendien om mogelijke infectiebronnen in beeld te krijgen en inzicht te geven op het voorkomen van infecties in de toekomst.

Gedurende het gehele onderzoek worden uw gegevens vertrouwelijk behandeld en aan de hand van uw antwoorden is uw identiteit niet te herleiden. Als blijk van waardering voor al uw inspanning ontvangt u een cadeaubon ter waarde van €20,- na het interview*.*

De vragenlijst heeft betrekking op de periode van uw reis, dus van 26 april tot en met 9 mei:
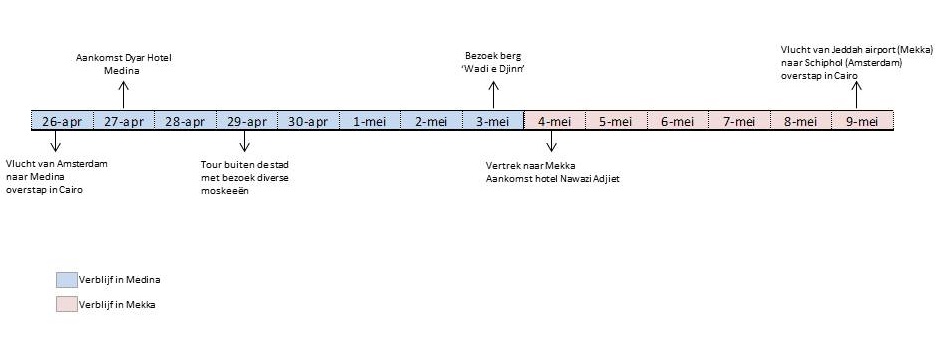


| **Uniek identificatienummer (in te vullen door de GGD-medewerker):** |
| --- |

A. Persoonsgegevens

A1. Geslacht ⬜ Man

⬜ Vrouw

A2. Geboortejaar ________(jjjj)

A3. Datum invullen vragenlijst ____(dd) / ____(mm) / ________(jjjj)

A4. Reden invullen vragenlijst:

U bent
⬜ Een patiënt met een door laboratoriumonderzoek aangetoonde MERS-CoV infectie

⬜ *In contact geweest* met een MERS-CoV patiënt.

A5. Hoeveel personen wonen in uw huishouden?

*Aantal invullen inclusief uzelf* A6.1 Kinderen onder de 18 jaar: ________
 A6.2 Volwassenen van 18 jaar en ouder: ________

B. Medische voorgeschiedenis

B1. Welke van onderstaande ziekten zijn ooit bij u vastgesteld door een huisarts of specialist?

*Meerdere antwoordopties mogelijk*

⬜ Suikerziekte / Diabetes Mellitus

⬜ Astma

⬜ COPD, emfyseem, chronische bronchitis

⬜ Overige longziekten, nl. ____________________________________________________________________

⬜ Hart- en vaatziekten, nl. ____________________________________________________________________

⬜ Afweerstoornis, nl. ________________________________________________________________________

⬜ Nierziekte, nl. ____________________________________________________________________________

⬜ Chronische leveraandoening zoals hepatitis, nl. _________________________________________________

⬜ Kwaadaardige ziekte (kanker), nl. _____________________________________________________________

⬜ Hematologische aandoening zoals chronische bloedarmoede, nl. ___________________________________

⬜ Allergie, nl. ______________________________________________________________________________

⬜ Andere ernstige aandoening, nl. ______________________________________________________________

⬜ Afgelopen drie maanden behandeld met immunoglobuline of bloedtransfusie gehad

⬜ Geen van bovenstaande

B2. Komt er een erfelijke aandoening voor in uw familie?

⬜ Ja

⬜ Nee

⬜ Onbekend

B2.1. Zo ja, welke erfelijke aandoening? ______________________________________________________

B4. **Enkel voor vrouwen:** bent u zwanger of bent u recent bevallen (afgelopen 6 maanden)? *(reden voor deze vraag is de vatbaarheid voor infecties)*

⬜ Ja

⬜ Nee

B5. Rookt u (wel eens)? *(reden voor deze vraag is de vatbaarheid voor infecties)*

⬜ Ja

⬜ Nee, maar vroeger wel

⬜ Nee, ik heb nooit gerookt


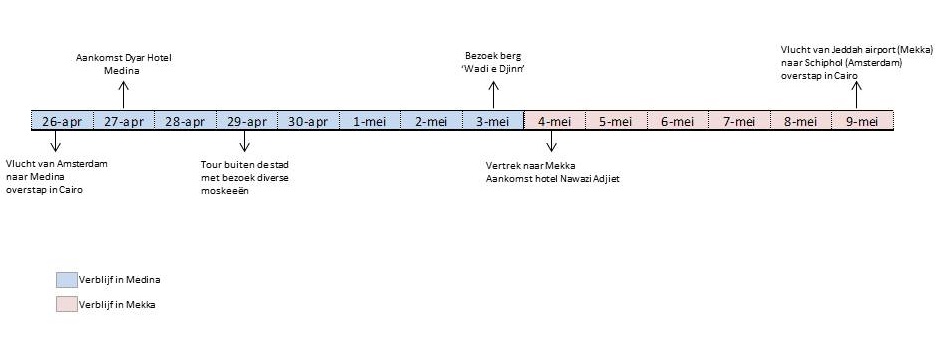
C. Blootstelling aan dieren

C1. Heeft u een locatie bezocht met levende dieren?

*Bijvoorbeeld geiten, schapen, dromedarissen, pluimvee, vogels op een markt, boerderij, kamelenrace, roofvogelshow, enz.*
⬜ Ja ⬜ Nee ⬜ Onbekend
Zo ja:
 C1.1 Waar en wanneer was dit *(volledig benoemen*)?

a. Waar:_____________________________________________Wanneer:____________________________

b. Waar:_____________________________________________Wanneer:____________________________

c. Waar:_____________________________________________Wanneer:____________________________

d. Waar:_____________________________________________Wanneer:____________________________

e. Waar:_____________________________________________Wanneer:____________________________

C1.2 Welke dieren heeft u daar gezien?

*Geef aan op welke locatie zoals bij vraag C1.1 genoemd, dit is geweest*

1. _____________________________________________________________________________________
2. _____________________________________________________________________________________
3. _____________________________________________________________________________________
4. _____________________________________________________________________________________
5. _____________________________________________________________________________________

C1.3 Heeft u contact gehad met deze dieren *(bijvoorbeeld aaien)*?

*Geef aan op welke locatie zoals bij vraag C1.1 genoemd, dit is geweest*

⬜ Ja ⬜ Nee ⬜ Onbekend

C1.3.1 Zo ja, met welke dieren?

1. _____________________________________________________________________________________
2. _____________________________________________________________________________________
3. _____________________________________________________________________________________
4. _____________________________________________________________________________________
5. _____________________________________________________________________________________


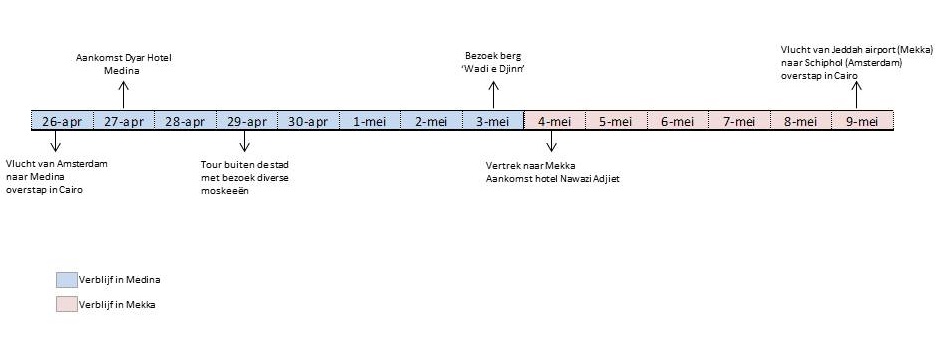


C1.4 Hebt u contact gehad met karkassen, lichaamsvloeistoffen, urine of ontlasting van dieren?

⬜ Ja ⬜ Nee ⬜ Onbekend

C1.4.1 Zo ja, wilt u dit nader toelichten?

a. Waar:_____________________________________________Wanneer:____________________________

b. Waar:_____________________________________________Wanneer:____________________________

c. Waar:_____________________________________________Wanneer:____________________________

d. Waar:_____________________________________________Wanneer:____________________________

e. Waar:_____________________________________________Wanneer:____________________________

C1.5 Hebt u voorwerpen aangeraakt die mogelijk in contact geweest zijn met dieren

*Bijvoorbeeld hekken, textiel, machines, stro, kleding)*?

⬜ Ja ⬜ Nee ⬜ Onbekend

C1.5.1 Zo ja, wilt u dit nader toelichten?

a. Waar:_____________________________________________Wanneer:____________________________

b. Waar:_____________________________________________Wanneer:____________________________

c. Waar:_____________________________________________Wanneer:____________________________

d. Waar:_____________________________________________Wanneer:____________________________

e. Waar:_____________________________________________Wanneer:____________________________

C1.6 Heeft u iets gegeten of gedronken terwijl u op een dierenmarkt/show was?

⬜ Ja ⬜ Nee ⬜ Onbekend

C1.6.1 Zo ja, wat, waar en wanneer?

a. Wat: __________________Waar:_______________________________Wanneer:___________________

b. Wat: __________________Waar:_______________________________Wanneer:___________________

c. Wat: __________________Waar:_______________________________Wanneer:___________________ d. Wat: __________________Waar:_______________________________Wanneer:___________________

e. Wat: __________________Waar:_______________________________Wanneer:___________________


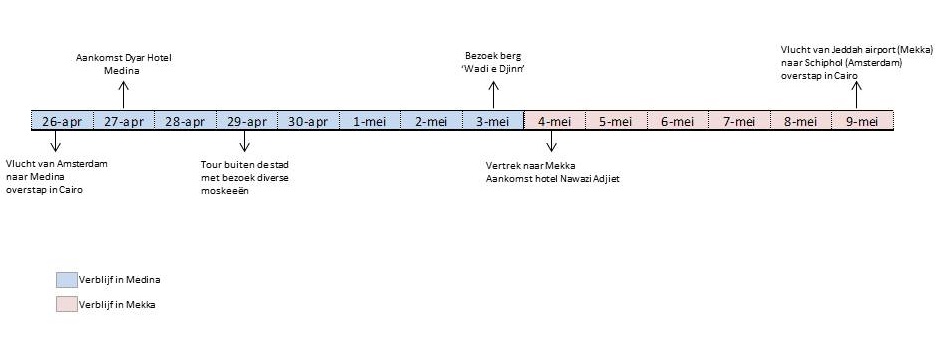
D. Blootstelling via voedsel of andere producten

D1. Heeft u rauwe melk gedronken?

⬜ Ja ⬜ Nee ⬜ Onbekend
D1.1 Zo ja, van welk dier? ______________________________________________________

D1.2 Waar? _________________________________________________________________

D1.3 Wanneer? ______________________________________________________________

D2. Heeft u producten gegeten die bereid zijn met rauwe melk?

⬜ Ja ⬜ Nee ⬜ Onbekend
D2.1 Zo ja, welke producten? ___________________________________________________

D2.2 Zo ja, van welk dier was de melk afkomstig? ___________________________________

D2.3 Waar? _________________________________________________________________

D2.4 Wanneer? ______________________________________________________________

D3. Heeft u een markt of kraampje bezocht*?*

⬜ Ja ⬜ Nee ⬜ Onbekend

Zo ja:

D3.1 Waar en wanneer was dit?

a. Waar:_____________________________________________Wanneer:____________________________

b. Waar:_____________________________________________Wanneer:____________________________

c. Waar:_____________________________________________Wanneer:____________________________

d. Waar:_____________________________________________Wanneer:____________________________

e. Waar:_____________________________________________Wanneer:____________________________

D3.2 Welke producten heeft u daar gegeten of gedronken?

*Bijvoorbeeld dierhuiden, vlees, melk, eieren, groente, fruit (gedroogd/vers), kruiden, vruchtensap, enz.*

1. _____________________________________________________________________________________
2. _____________________________________________________________________________________
3. _____________________________________________________________________________________
4. _____________________________________________________________________________________
5. _____________________________________________________________________________________

D4. Heeft u souvenirs gekocht op een markt of stalletje?

⬜ Ja ⬜ Nee ⬜ Onbekend
 D4.1 Zo ja, wat, waar en wanneer?

a. Wat: __________________Waar:_______________________________Wanneer:___________________

b. Wat: __________________Waar:_______________________________Wanneer:___________________

c. Wat: __________________Waar:_______________________________Wanneer:___________________ d. Wat: __________________Waar:_______________________________Wanneer:___________________

e. Wat: __________________Waar:_______________________________Wanneer:___________________


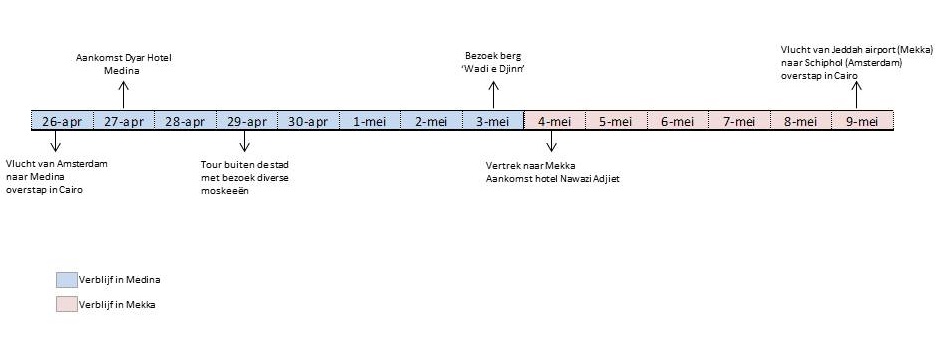
E. Blootstelling via mensen

E1. Heeft u contact gehad met een persoon met een luchtweginfectie met hoesten en koorts?
⬜ Ja ⬜ Nee ⬜ Onbekend
Zo ja:
 E1.1 Wat is u relatie ten opzichte van deze persoon? __________________________________________
 E1.2 Waar vond het contact plaats?________________________________________________________

E1.3 Wanneer? ________________________________________________________________________

E2. Heeft u een ziekenhuis bezocht als patiënt?

⬜ Ja ⬜ Nee ⬜ Onbekend
Zo ja:
 E2.1 Wat is de naam en locatie van het ziekenhuis? ___________________________________________
 E2.2 Wat was de reden van het ziekenhuisbezoek? ___________________________________________
 E2.3 Hoe lang hebt u in de wachtkamer doorgebracht? ________________________________________

E2.4 Wanneer was u daar? _______________________________________________________________
 E2.5 Bent u hier in contact geweest met patiënten met een luchtweginfectie? ⬜ Ja ⬜ Nee ⬜ Onbekend

E3. Heeft u een ziekenhuis bezocht als begeleider van een patiënt?

⬜ Ja ⬜ Nee ⬜ Onbekend
Zo ja:
 E3.1 Wat is de naam en locatie van het ziekenhuis? ___________________________________________
 E3.2 Hoe lang hebt u in de wachtkamer doorgebracht? ________________________________________

E3.3 Wanneer was u daar? _______________________________________________________________
 E3.4 Bent u hier in contact geweest met patiënten met een luchtweginfectie? ⬜ Ja ⬜ Nee ⬜ Onbekend

E4. Heeft u een traditioneel genezer bezocht?

⬜ Ja ⬜ Nee ⬜ Onbekend
Zo ja:
 E4.1 Wat is de naam en het adres van deze arts? _____________________________________________
 E4.2 Wat was de reden van het bezoek aan deze arts? _________________________________________

E4.3 Wanneer was dat? _________________________________________________________________
 E4.4 Heeft de arts medicijnen voorgeschreven? ⬜ Ja ⬜ Nee ⬜ Onbekend
 E4.4.1 Zo ja, welke? __________________________________________________________________

E5. Heeft u direct contact gehad met de patiënt tijdens zijn/haar ziekte?
⬜ Ja ⬜ Nee ⬜ Onbekend
 E5.1 Zo ja, wat voor soort contact was dit? ________________________________________________

E5.2 Wanneer was dat? ________________________________________________________________

E6. Heeft u contact gehad met urine, bloed, sputum of ontlasting van de patiënt?
⬜ Ja ⬜ Nee ⬜ Onbekend
 E6.1 Zo ja, welke vloeistof was dit? _________________________________________________________

E6.2 Wanneer was dat? __________________________________________________________________

E7. Heeft u in de week voorafgaand aan uw reis contact gehad met mensen die net teruggekeerd waren uit Saoedi-Arabië?

*Bijvoorbeeld via moskee of groepsbijeenkomsten*

⬜ Ja ⬜ Nee ⬜ Onbekend

E5.1 Zo ja, waar was dit? _______________________________________________________________

E5.2 Wanneer was dit? ________________________________________________________________

Mogen we u bij het vervolg van het onderzoek nog benaderen voor extra informatie?

⬜ Ja ⬜ Nee

Zo ja:

Hoe mogen we contact met u opnemen?

⬜ Per telefoon, telefoonnummer_______________________________________________________________

⬜ Per e-mail _______________________________________________________________________________

Heeft u nog opmerkingen?

_________________________________________________________________________________________________________________________________________________________________________________________________________________________________________________________________________________

**Hartelijk bedankt voor uw medewerking aan deze vragenlijst.**
